# Efficacy and Safety of a Bioinspired Chitosan–Catechol/Gelatin Hemostatic Patch vs. TachoSil in Hepatectomy: A Randomized Noninferiority Trial

**DOI:** 10.3390/biomedicines14051087

**Published:** 2026-05-12

**Authors:** Seoung Hoon Kim, Keumyeon Kim, Kyoungok Yun, Gyu-Seong Choi

**Affiliations:** 1 Organ Transplantation Center, National Cancer Center, 323 Ilsan-ro, Ilsandong-gu, Goyang-si 10408, Gyeonggi-do, Republic of Korea; kshlj@ncc.re.kr; 2 Medical Device Division, SCL Science Inc., 25, Seonyu-ro 13-gil, Yeongdeungpo-gu, Seoul 07282, Republic of Korea; keumyeon_kim@sclscience.com (K.K.); koyoon@sclscience.com (K.Y.); 3 Department of Surgery, Samsung Medical Center, Sungkyunkwan University School of Medicine, 81, Irwon-ro, Gangnam-gu, Seoul 06351, Republic of Korea

**Keywords:** hemostatic biomaterial, bioinspired adhesive, chitosan–catechol, gelatin, hemostasis, hepatectomy, surgical bleeding, translational biomaterial, randomized controlled trial

## Abstract

**Background/Objectives**: Topical hemostatic biomaterials are used to control diffuse parenchymal bleeding during hepatectomy. TachoSil is a widely used standard fibrin sealant patch. We evaluated the efficacy and safety of InnoSEAL Plus DL, a novel bioinspired absorbable chitosan–catechol/gelatin hemostatic patch, compared with TachoSil. **Methods**: This multicenter, randomized, single-blind, active-controlled, parallel-group noninferiority trial enrolled adults undergoing hepatectomy who had persistent oozing from the hepatic transection surface despite primary hemostasis. Participants were randomized in a 1:1 ratio to receive InnoSEAL Plus DL or TachoSil. The primary endpoint was hemostatic success within 3 min of application, with a prespecified noninferiority margin of −19.4 percentage points (pp). Safety was assessed up to 30 days postoperatively. **Results**: Ninety patients were randomized (45 per group). In the per-protocol population, 3 min hemostatic success was achieved in 100.0% of both the InnoSEAL Plus DL (43/43) and TachoSil (41/41) groups. The risk difference was 0.0 pp, and the lower bound of the one-sided 97.5% confidence interval was −8.2 pp, confirming noninferiority. The mean time to hemostasis was similar between groups (1.2 vs. 1.3 min), and no intraoperative rebleeding occurred. Adverse events were reported in 78/90 patients (86.7%) and serious adverse events in 6/90 (6.7%); the latter were typical post-hepatectomy events unrelated to the study devices. No deaths were reported. **Conclusions**: InnoSEAL Plus DL was noninferior to TachoSil for achieving rapid intraoperative hemostasis during hepatectomy, with no unexpected safety concerns. This bioinspired hemostatic patch is an effective alternative to fibrin sealant, without the use of human-derived proteins.

## 1. Introduction

Topical hemostatic biomaterials are increasingly recognized as important adjuncts in the management of surgical bleeding, particularly in friable and highly vascularized tissues [1,2,3]. Among solid organs, the liver presents a uniquely challenging hemostatic environment because its dense microvascular structure, fragile parenchyma, and limited vasoconstrictive capacity predispose it to persistent diffuse oozing [4,5]. Hepatectomy remains a cornerstone treatment for hepatocellular carcinoma and other hepatic diseases [6,7]. However, despite major advances in surgical techniques, imaging, and perioperative care, intraoperative and postoperative bleeding continue to substantially influence perioperative outcomes [8,9,10]. Although post-hepatectomy hemorrhage is relatively uncommon, with a reported incidence of 1–8%, it remains a serious complication, as it contributes significantly to both postoperative morbidity and mortality [7,10,11]. Furthermore, intraoperative blood loss and perioperative transfusion have been independently associated with prolonged hospitalization, increased complication rates, and impaired long-term oncological outcomes following hepatectomy, underscoring the clinical importance of meticulous intraoperative hemostasis [12,13,14].

Even with refinements in anesthetic management, low central venous pressure techniques, and modern parenchymal transection devices, diffuse oozing from the hepatic transection surface remains a frequent intraoperative challenge that conventional surgical techniques alone cannot fully resolve [4,8,12]. This challenge is further amplified in patients with chronic liver disease, in whom impaired hepatic reserve, coagulopathy, and rebalanced but fragile hemostasis may complicate bleeding control [7,8,15,16,17,18]. In such settings, conventional measures, including suturing, ligation, and electrocautery, are often insufficient or impractical for broad oozing surfaces after parenchymal transection [1,4,5,19,20,21]. Accordingly, there remains an unmet clinical need for topical hemostatic materials that provide rapid and effective hemostasis, conform to irregular wet-tissue surfaces, and perform reliably even in challenging operative conditions.

Topical hemostatic agents are generally classified as passive or active based on their mode of action [2,22]. Passive agents, such as gelatin-, collagen-, and cellulose-based materials, primarily provide a structural matrix that facilitates endogenous clot formation, whereas active agents contain thrombin and fibrinogen to directly accelerate hemostasis [2,22]. TachoSil (Corza Medical, Linz, Austria), a fibrin sealant-coated equine collagen patch containing human thrombin and fibrinogen, has been widely used in hepatic surgery because of its favorable handling characteristics and extensive clinical experience [1,8]. Nevertheless, fibrin sealant patches depend on exogenous biologically active coagulation components derived from human or animal plasma, which raises ongoing considerations regarding theoretical pathogen transmission, immunogenicity upon repeated exposure, and vulnerabilities in the plasma supply chain [23,24]. These considerations underscore the need for alternative biomaterial platforms that can reduce the risks associated with biologic components while maintaining or improving hemostatic performance [1,25].

Bioinspired adhesive biomaterials have emerged as promising translational strategies for surgical hemostasis because their capacity to adhere to wet-tissue surfaces offers practical advantages in clinical settings [26,27]. Mussel foot proteins achieve remarkable wet adhesion through 3,4-dihydroxyphenyl-L-alanine (DOPA) residues [28,29]. The catechol moiety of DOPA enables both covalent and noncovalent interactions with biological tissues, facilitating robust adhesion under aqueous conditions [29,30,31]. Building on this principle, mussel-inspired catechol chemistry has been extensively investigated as a strategy to enhance interfacial adhesion under wet physiological conditions and has been applied to the development of biomedical adhesives and hemostatic materials [26,27,32,33,34]. Catechol-functionalized chitosan (CHI-C) is designed to mimic mussel-inspired interfacial chemistry and can rapidly interact with blood proteins and cellular components to generate a blood-insoluble adhesive barrier [34,35]. Unlike conventional coagulation factor-based systems, this mechanism supports hemostasis through wet-tissue adhesion, physical sealing, and barrier formation in a manner that is not primarily dependent on the intact coagulation cascade [35]. CHI-C has demonstrated effective hemostatic performance in animal models and in a first-in-human study [35]. Furthermore, a subsequent preclinical study using an anticoagulated swine model of gastrointestinal bleeding showed rapid hemostasis coupled with favorable tissue healing responses [36]. Taken together, these findings support the translational potential of catechol-based hemostatic biomaterials in clinical settings involving surgical bleeding.

Based on these findings, InnoSEAL Plus DL (SCL Science Inc., Seoul, Republic of Korea) was developed as an absorbable double-layer hemostatic patch composed of a CHI-C adhesive layer and a gelatin sealing layer [37]. The CHI-C layer promotes wet-tissue adhesion and hemostasis through interactions with blood proteins, whereas the gelatin layer contributes absorptive sealing and mechanical support [37]. Given these properties, InnoSEAL Plus DL was developed as a clinically relevant biomaterial-based alternative to fibrin sealants for hepatic parenchymal bleeding.

Therefore, we conducted a multicenter, randomized, single-blind, active-controlled, parallel-group noninferiority trial comparing InnoSEAL Plus DL with TachoSil in adults undergoing hepatectomy who exhibited persistent oozing despite primary hemostasis. The primary objective was to determine whether InnoSEAL Plus DL was noninferior to TachoSil in achieving hemostatic success within 3 min after device application, while also evaluating its safety profile.

## 2. Materials and Methods

### 2.1. Study Design and Participants

This was a multicenter, randomized, single-blind, active-controlled, parallel-group noninferiority trial comparing InnoSEAL Plus DL with TachoSil for persistent oozing from the hepatic transection surface despite primary hemostasis during hepatectomy. The trial was conducted at Samsung Medical Center (Seoul, Republic of Korea) and the National Cancer Center (Goyang-si, Republic of Korea). The protocol was approved by the Ministry of Food and Drug Safety (MFDS) of the Republic of Korea and by the institutional review boards of both participating centers. No important changes were made to the methods after trial commencement. The trial registered with the Clinical Research Information Service (CRIS; http://cris.nih.go.kr; accessed on 27 March 2026; Identifier: KCT0011267).

Adults scheduled to undergo hepatectomy were screened preoperatively. Potential participants were identified through the Department of Hepatobiliary Surgery and the Liver Transplantation Center and were referred by their treating surgeons. Written informed consent was obtained from all patients before screening and any study-specific procedures. Bleeding severity at the hepatic transection surface was classified using prespecified protocol criteria. Eligible bleeding was limited to oozing bleeding, defined as clinically silent, minor capillary bleeding from small vessels that does not require additional invasive hemostatic procedures [38,39,40,41]. Major bleeding (an exclusion criterion) was defined as spurting hemorrhage (typically arterial) or bleeding from major hepatic veins draining into the inferior vena cava [38,42,43]. Patients were eligible if they were aged ≥ 19 years, provided written informed consent, and had persistent oozing from the hepatic transection surface after completion of hepatectomy despite standard primary hemostatic measures. Key exclusion criteria included major bleeding after primary hemostasis, severe coagulopathy, severe hepatic dysfunction, hypersensitivity to study device components, pregnancy or breastfeeding, emergency hepatectomy, and any condition judged by the investigator to preclude safe participation. Patients with major bleeding after primary hemostasis were excluded on clinical grounds because such bleeding requires immediate definitive surgical management and is outside the intended adjunctive-use setting of the study devices. Therefore, the trial appropriately restricted randomization to patients with persistent oozing after primary hemostasis, for whom adjunctive treatment with an absorbable topical hemostatic patch was considered clinically appropriate. Full eligibility criteria are provided in Supplementary Table S1.

### 2.2. Randomization and Blinding

After intraoperative confirmation of eligibility, participants were randomized 1:1 to InnoSEAL Plus DL or TachoSil by a center-stratified permuted-block randomization schedule generated by a statistician not involved in patient care using PROC PLAN in SAS software (version 9.4; SAS Institute Inc., Cary, NC, USA). Allocation was concealed using sequentially numbered, opaque, sealed envelopes prepared according to the randomization schedule. These were opened only after the operating surgeon had directly visualized the bleeding severity at the hepatic transection surface and confirmed that it met the oozing definition and did not meet that of major bleeding. Therefore, the assigned device could not influence the eligibility adjudication. Because the study device had to be applied intraoperatively, surgeons and operating-room staff were unblinded, whereas participants remained blinded to treatment allocation. To preserve analytical blinding, the statistician who generated the randomization schedule was different from the one who performed the final analyses.

### 2.3. Trial Procedures and Interventions

Participants underwent open or laparoscopic hepatectomy according to standard practice at each center. After hepatectomy, primary hemostasis of the transection surface was achieved using conventional measures, including suturing, ligation, vascular clips, argon beam coagulation, and electrocautery. Patients were randomized only if persistent oozing remained and major bleeding was absent after primary hemostasis.

The device was applied according to a prespecified protocol and the respective instructions for use (IFU). For each target bleeding site, the allocated device was trimmed to extend approximately 1–2 cm beyond the bleeding area to ensure complete coverage. The assigned contact surface was then applied directly to the hepatic transection surface: the CHI-C adhesive layer (blue) for InnoSEAL Plus DL (SCL Science Inc., Seoul, Republic of Korea) and the active-coated surface (yellow) for TachoSil (Corza Medical, Linz, Austria). The device was manually compressed against the bleeding surface for 3 min. In open surgery, compression was applied directly over the device. In laparoscopic surgery, the device was introduced through the trocar port, unfolded to cover the target bleeding site, and compressed using a grasper for the same 3 min period.

Hemostasis at the target bleeding site was assessed visually at prespecified time points (3, 4, 5, 8, 9, and 10 min) after device application. Hemostatic success was defined as the absence of visible active bleeding at the target bleeding site after application of the study device. Objective visual criteria included no bleeding from the edges of the applied device and no bleeding through the device. If either finding was observed, hemostasis was not considered achieved, and assessment continued according to the prespecified time points. If persistent oozing remained at 10 min after device application, treatment was considered a failure, and additional hemostatic measures were permitted at the surgeon’s discretion. Before abdominal closure, the treated site was reassessed for rebleeding. Postoperative safety evaluations were performed through postoperative day (POD) 30.

### 2.4. Outcomes

The primary endpoint was hemostatic success within 3 min after device application, and secondary endpoints were hemostatic success within 10 min, time to hemostasis, and intraoperative rebleeding at the treated site before abdominal closure. Safety endpoints included treatment-emergent adverse events (TEAEs), serious adverse events (SAEs), vital signs, and routine laboratory parameters. Adverse events (AEs) were coded using the Medical Dictionary for Regulatory Activities (MedDRA, version 26.1) and were assessed for severity and relationship to the study device.

### 2.5. Sample Size

Sample size was determined for the primary endpoint of hemostatic success within 3 min after device application using a noninferiority design on the absolute risk difference scale. The noninferiority margin was prespecified using a conservative fixed margin (M_1_/M_2_) framework with 50% effect retention, consistent with principles described in the FDA Guidance [44].

During trial planning, historical evidence supporting the hemostatic efficacy of TachoSil in hepatic surgery was reviewed. In the FDA Statistical Review for TachoSil, pooled pivotal hepatic studies reported 3 min hemostatic success rates of 74.7% for TachoSil and 50.6% for the comparator treatment [45]. Therefore, the comparator success rate of 50.6% was used as a regulatorily evaluated benchmark. For the assumed 3 min hemostatic success rate of TachoSil in the present trial, we used 89.31%, corresponding to a conservative lower 95% confidence bound for the 3 min hemostatic success rate observed in the TachoSil group (48/49; 97.96%) of our previously published multicenter randomized noninferiority trial comparing InnoSEAL Plus, a single-layer CHI-C-based hemostatic patch, with TachoSil in hepatectomy patients [38]. Because the prior study was conducted at the same institutions and by the same investigator group as the present trial, it provided a directly relevant estimate of the expected active control performance.

Based on these assumptions, the estimated active control effect was:

$$M_{1}=\;89.31\%\;-\;50.6\%\;=\;38.71\;pp$$

To preserve at least 50% of this effect, the noninferiority margin (M_2_) was set as:

$$M_{2}=38.71\;pp\times 0.5=19.36\;pp\approx 19.4\;pp$$

Because the primary analysis was expressed as the absolute risk difference between InnoSEAL Plus DL and TachoSil, the noninferiority margin was specified as −19.4 pp. Noninferiority was considered demonstrated if the lower bound of the one-sided 97.5% confidence interval (CI) for the absolute risk difference was greater than or equal to −19.4 pp.

Assuming a one-sided alpha level of 0.025, 80% power, and 1:1 allocation, 40 participants were required per group. Allowing for a 10% dropout rate, the target enrollment was 45 participants per group (*N* = 90).

### 2.6. Statistical Analysis

Statistical analyses were prespecified in the statistical analysis plan and performed using SAS software (version 9.4; SAS Institute Inc., Cary, NC, USA). Efficacy was analyzed in both the per-protocol (PP) and intention-to-treat (ITT) populations. The latter corresponded to the protocol-defined full analysis set (FAS) and included all randomized participants who received the assigned device and underwent efficacy assessment. The former was the primary analysis set for the noninferiority analysis, and the ITT population was used for supportive sensitivity analyses. The safety population included all participants who received a study device.

The primary endpoint, hemostatic success within 3 min after device application, was analyzed on the absolute risk difference scale (InnoSEAL Plus DL minus TachoSil). Noninferiority was concluded if the lower bound of the one-sided 97.5% CI for the risk difference was greater than or equal to the prespecified margin of −19.4 pp. The two-sided 95% CI for the risk difference was calculated using the Newcombe–Wilson score method, such that the one-sided 97.5% lower confidence bound corresponded to the lower limit of the two-sided 95% CI. The two-sided 95% CI for the risk ratio was calculated using the exact binomial (Clopper–Pearson) method. Secondary and safety endpoints were compared using the independent two-sample *t*-test or Wilcoxon rank-sum test for continuous variables and the chi-square test or Fisher’s exact test for categorical variables, as appropriate. Except for the primary noninferiority test, all tests were two-sided with a significance level of 0.05. No imputation was performed for missing data.

## 3. Results

### 3.1. Patient Selection and Analysis Populations

**Figure 1:**
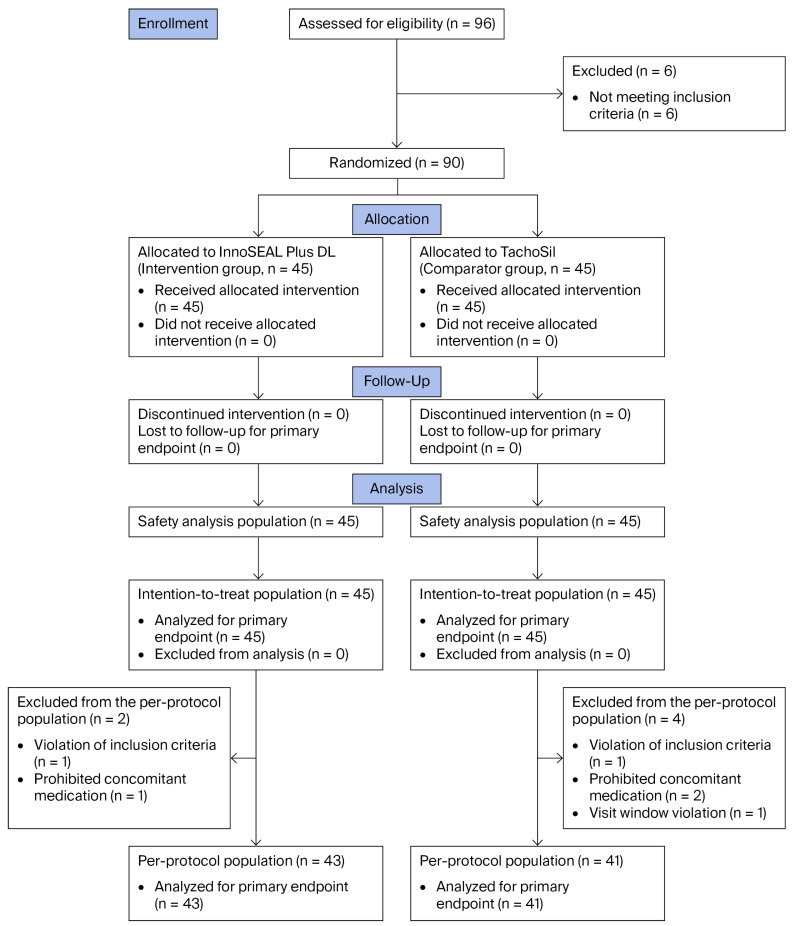
CONSORT flow diagram of participant disposition.

From June 2022 to January 2024, a total of 96 patients were screened at the two study centers (Figure 1), 6 of whom were excluded before randomization due to not meeting the inclusion criteria. The remaining 90 patients were randomly assigned in a 1:1 ratio to the intervention group (InnoSEAL Plus DL, *n* = 45) or the comparator group (TachoSil, *n* = 45), and all received the allocated intervention. Patients were evaluated for safety at postoperative day (POD) 7 and POD 30, either during hospitalization or at scheduled outpatient visits. No patient discontinued the allocated intervention or was lost to follow-up for the primary endpoint; therefore, all 90 treated patients were included in the safety population and constituted the intention-to-treat (ITT) population (InnoSEAL Plus DL, *n* = 45; TachoSil, *n* = 45). For the per-protocol (PP) analysis, two patients in the InnoSEAL Plus DL group and four patients in the TachoSil group were excluded because of major protocol deviations. These deviations consisted of eligibility criteria violations in two patients, use of prohibited concomitant medication in three patients, and a visit window violation in one patient. The three patients with prohibited concomitant medication received tranexamic acid, an antifibrinolytic agent, for the treatment of postoperative adverse events (AEs). These cases included one patient in the InnoSEAL Plus DL group and two patients in the TachoSil group. Because tranexamic acid could theoretically affect hemostatic outcomes, these cases were classified as major protocol deviations and excluded from the PP population. The PP population therefore comprised 84 patients (InnoSEAL Plus DL, *n* = 43; TachoSil, *n* = 41), all of whom were included in the primary endpoint analysis.

### 3.2. Patient Characteristics

Baseline demographic and clinical characteristics of the ITT population are summarized in Table 1. Overall, baseline characteristics were generally similar between the two groups. The mean age was 46.5 ± 15.3 years in the InnoSEAL Plus DL group and 48.2 ± 13.0 years in the TachoSil group, and sex distribution was comparable between groups (26 [57.8%] vs. 21 [46.7%] males, respectively). Height and weight were also similar between groups.

The most common indications for hepatectomy were donor hepatectomy and hepatocellular carcinoma in both groups. The latter was more frequent in the InnoSEAL Plus DL group (19 [42.2%] vs. 15 [33.3%]), whereas the former was more frequent in the TachoSil group (22 [48.9%] vs. 19 [42.2%]). Underlying liver disease and comorbid conditions were also similar between the groups. Hepatitis B was present in 14 (31.1%) vs. 12 (26.7%), cirrhosis in 6 (13.3%) vs. 10 (22.2%), hypertension in 11 (24.4%) vs. 13 (28.9%), and diabetes mellitus in 8 (17.8%) vs. 5 (11.1%) patients in the InnoSEAL Plus DL and TachoSil groups, respectively. In accordance with CONSORT recommendations, no formal hypothesis testing of between-group differences for baseline characteristics was performed.

### 3.3. Procedural Characteristics

Procedural characteristics of the ITT population are summarized in Table 2. Of the 90 procedures, 66 (73.3%) were performed at Samsung Medical Center and 24 (26.7%) at the National Cancer Center.

Overall, the surgical approach, hepatic parenchymal morphology, resection type and primary hemostatic techniques were generally similar between the two groups. Laparoscopic hepatectomy was the predominant surgical approach in both groups (32 [71.1%] in the InnoSEAL Plus DL group vs. 33 [73.3%] in the TachoSil group), whereas open hepatectomy was performed in 13 (28.9%) vs. 12 (26.7%) patients, respectively. Most patients had normal liver morphology, and all instances of abnormal morphology were cirrhosis (8 [17.8%] vs. 7 [15.6%]). Hemihepatectomy was the most common resection type in both groups (27 [60.0%] vs. 31 [68.9%]). Primary hemostasis was achieved using cautery in all patients, frequently accompanied by adjunctive sutures/ligation (43 [95.6%] vs. 45 [100.0%]) or vascular clips (32 [71.1%] vs. 33 [73.3%]). These techniques were not mutually exclusive.

Concomitant non-study procedures, defined as non-drug treatments administered after application of the assigned device, were reported in 13/90 patients (14.4%) and were more frequent in the InnoSEAL Plus DL group (11/45 [24.4%], 16 procedures) than in the TachoSil group (2/45 [4.4%], 4 procedures). The most frequently reported concomitant procedure was thoracic cavity drainage (5/45 [11.1%] vs. 1/45 [2.2%]), followed by transfusion (2/45 [4.4%] vs. 2/45 [4.4%]). Other procedures were infrequent and included platelet transfusion, abscess drainage, bladder catheterization, oxygen therapy, red blood cell transfusion, and wound treatment. These concomitant procedures were mainly performed for postoperative clinical management, including treatment or prevention of hepatectomy-related AEs. Concomitant medications were reported in 87/90 (96.7%) patients, with similar use between groups (44/45 [97.8%] with 344 records vs. 43/45 [95.6%] with 262 records, respectively). Three patients (one in the InnoSEAL Plus DL group and two in the TachoSil group) received tranexamic acid, a prohibited antifibrinolytic agent, to treat AEs. Because tranexamic acid could theoretically influence hemostatic outcomes by inhibiting fibrinolysis, these cases were classified as major protocol deviations, leading to exclusion from the PP population (Figure 1).

### 3.4. Primary Endpoint

In the PP population, the primary endpoint of hemostatic success within 3 min after device application on the hepatic transection surface was achieved in all patients in both groups (43/43 [100.0%] in the InnoSEAL Plus DL group vs. 41/41 [100.0%] in the TachoSil group; Table 3). The absolute risk difference for 3 min hemostatic success (InnoSEAL Plus DL minus TachoSil) was 0.0 pp, with a two-sided 95% CI of −8.2 to 8.6 pp (Table 3). The lower bound (−8.2 pp), equivalent to the one-sided 97.5% lower confidence bound, was above the prespecified noninferiority margin of −19.4 pp, demonstrating that InnoSEAL Plus DL was noninferior to TachoSil. The corresponding risk ratio was 1.0 (95% CI, 0.9 to 1.1).

In the ITT population, hemostatic success within 3 min was also 100.0% in both groups (45/45 [100.0%] in each group; Table 3), with an absolute risk difference of 0.0 pp (two-sided 95% CI, −7.9 to 7.9 pp) and a risk ratio of 1.0 (95% CI, 0.9 to 1.1), supporting the noninferiority conclusion. Because primary endpoint data were available for all patients in both analysis populations, no imputation for missing data was required. The ITT analysis, prespecified as a supportive/sensitivity analysis, yielded results consistent with those of the PP analysis.

### 3.5. Secondary Endpoints

Secondary endpoints for the PP and ITT populations are summarized in Table 3. In the PP population, hemostatic success within 10 min was achieved in all patients in both groups (43/43 [100.0%] in the InnoSEAL Plus DL group vs. 41/41 [100.0%] in the TachoSil group). The absolute risk difference for hemostatic success within 10 min (InnoSEAL Plus DL minus TachoSil) was 0.0 pp (95% CI, −8.2 to 8.6 pp), with a risk ratio of 1.0 (95% CI, 0.9 to 1.1). Similar results were observed in the ITT population, in which hemostatic success within 10 min was also 100.0% in both groups (45/45 [100.0%] in each group), with an absolute risk difference of 0.0 pp (95% CI, −7.9 to 7.9 pp) and a risk ratio of 1.0 (95% CI, 0.9 to 1.1).

The time to hemostasis was short and comparable between groups. In the PP population, the mean time to hemostasis was 1.2 ± 0.4 min in the InnoSEAL Plus DL group vs. 1.3 ± 0.4 min in the TachoSil group, with median values of 1.1 min (range, 0.8–3.0) and 1.1 min (range, 1.0–3.0), respectively (*p* = 0.979). In the ITT population, the corresponding mean values were 1.2 ± 0.4 vs. 1.3 ± 0.4 min, with median times of 1.1 min (range, 0.8–3.0) and 1.1 min (range, 1.0–3.0), respectively (*p* = 0.942).

No rebleeding was observed in either treatment group in either analysis population. Therefore, formal hypothesis testing and effect estimation for rebleeding were not performed. No prespecified subgroup analyses were performed.

### 3.6. Safety

Safety outcomes in the safety population are summarized in Table 4. From device application through the end of follow-up, at least one adverse event (AE) was reported in 78/90 patients (86.7%), with 193 events in total. The proportion of patients with at least one AE was identical between groups (39/45 [86.7%] in each group; *p* = 1.000), although the total number of AEs was higher in the InnoSEAL Plus DL group than in the TachoSil group (110 vs. 83 events). Most AEs were mild or moderate: mild AEs were reported in 33/45 patients (73.3%) in the InnoSEAL Plus DL group and 28/45 (62.2%) in the TachoSil group, whereas moderate AEs were reported in 16/45 (35.6%) and 14/45 (31.1%), respectively. Severe AEs were uncommon and occurred in 2/45 patients (4.4%) in each group. No device-related AEs or deaths were reported. The most common AE categories, including gastrointestinal symptoms, procedural pain, respiratory or thoracic events, and transient postoperative findings, were clinically consistent with events commonly encountered after hepatectomy rather than suggestive of a device-specific toxicity signal.

Serious adverse events (SAEs) occurred in 6/90 patients (6.7%), with six events in total. SAEs occurred in 4/45 patients (8.9%) in the InnoSEAL Plus DL group and 2/45 (4.4%) in the TachoSil group (*p* = 0.677), with four and two events reported, respectively. Pleural effusion was the most frequently reported SAE, occurring in 4/45 patients (8.9%) in the InnoSEAL Plus DL group vs. 1/45 (2.2%) in the TachoSil group, whereas intra-abdominal hematoma occurred in 1 patient (2.2%) in the TachoSil group only. These events are clinically recognized postoperative complications after liver resection and were managed with standard postoperative treatment. Most SAEs were moderate in severity, but one severe SAE was reported in the TachoSil group. All patients with SAEs recovered without sequelae, and no device-related SAEs or deaths were observed. Concomitant non-study procedures were more frequent in the InnoSEAL Plus DL group and were mainly performed for postoperative clinical management, including treatment or prevention of hepatectomy-related AEs. This imbalance should be considered when interpreting the higher total number of AEs and SAEs in the InnoSEAL Plus DL group; however, no device-related AEs, device-related SAEs, or deaths occurred, and all patients with SAEs recovered without sequelae. Overall, the observed safety profile was consistent with events commonly encountered after liver resection, and no unexpected or clinically meaningful device-related safety concern was identified.

## 4. Discussion

In this multicenter, randomized, single-blind, active-controlled, parallel-group noninferiority trial, we found that InnoSEAL Plus DL, a bioinspired absorbable bilayer hemostatic biomaterial composed of chitosan–catechol (CHI-C) and gelatin, demonstrated clinical performance comparable to that of the established fibrin sealant patch TachoSil for the control of parenchymal oozing during hepatectomy. Noninferiority was demonstrated for the primary endpoint of 3 min hemostatic success; secondary hemostatic outcomes were similar between groups, and no unexpected device-related safety concerns were observed. These findings extend prior preclinical and early clinical observations of CHI-C and provide translational clinical evidence supporting this biomaterial platform in a demanding surgical field [34,35,36,37,38].

The present findings should be interpreted within the evolving landscape of surgical hemostatic biomaterials. Most conventional topical agents act primarily by providing a structural matrix that promotes endogenous clot formation or by delivering exogenous coagulation factors such as fibrinogen and thrombin to accelerate the final steps of the coagulation cascade [2,22,23]. In contrast, bioinspired adhesive systems are designed to address an additional and clinically important requirement, namely, the establishment of stable interfacial contact between the biomaterial and the wet, dynamic bleeding tissue surface, which is essential for sustained hemostatic performance under physiological conditions [26,27,30,31,32,33,34,35,36,37,38]. From this perspective, the comparable hemostatic performance of InnoSEAL Plus DL and TachoSil suggests that a non-fibrin, catechol-functionalized biopolymer patch can achieve clinically meaningful hemostasis through a design rationale distinct from that of fibrinogen/thrombin-dependent fibrin sealants [35,37,46].

These findings are clinically relevant in the context of hepatectomy, in which effective control of bleeding from the transection surface remains essential for reducing perioperative morbidity [4,8,9,10,11]. Although advances in operative technique and perioperative care have improved outcomes, persistent parenchymal oozing despite primary hemostasis remains a common intraoperative challenge [4,8,9,10]. This issue is particularly critical in patients undergoing hepatectomy in the setting of chronic liver disease, as impaired hepatic function compromises coagulation and thereby increases the importance of effective hemostatic control [15,16,17,18]. Therefore, topical hemostatic agents are widely used as adjuncts when conventional measures alone are insufficient or impractical [1,2,3,4,22]. However, previous systematic reviews [19,21] and randomized studies [39,40,41] have also suggested that the clinical benefit of topical hemostatic agents may vary depending on the type of agent, bleeding severity, surgical setting, and outcome assessed. This variability underscores the importance of evaluating new hemostatic biomaterials in well-defined clinical scenarios with standardized endpoints [19,21,39,40,41]. Against this background, the present trial shows that InnoSEAL Plus DL can achieve rapid hemostasis comparable to that of an established fibrin sealant patch in a well-defined and clinically relevant intraoperative setting.

**Figure 2:**
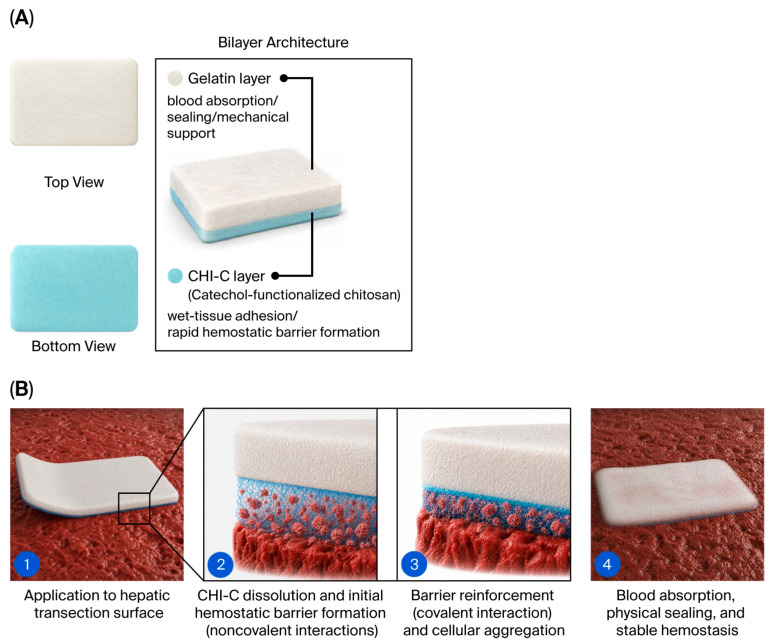
Schematic illustration of the structural architecture and proposed hemostatic mechanism of InnoSEAL Plus DL. (**A**) The bilayer architecture of InnoSEAL Plus DL. The patch consists of a gelatin layer, which absorbs blood, provides physical sealing, and supports mechanical reinforcement, and a CHI-C layer that mediates wet-tissue adhesion and the rapid formation of a blood-insoluble hemostatic barrier. The top and bottom views illustrate the visual appearance of the gelatin (white) and CHI-C (blue) layers, respectively. (**B**) The proposed hemostatic mechanism on the hepatic transection surface. ① InnoSEAL Plus DL is applied to the hepatic transection surface with the CHI-C layer in direct contact with the bleeding tissue. ② Upon contact with blood, the CHI-C layer rapidly dissolves and binds blood proteins through noncovalent catechol-mediated interactions, forming an initial blood-insoluble hemostatic barrier. ③ Subsequent covalent bonding between catechol groups of CHI-C and tissue and blood proteins, together with chitosan-mediated aggregation of platelets and blood cells, reinforces the hemostatic barrier. ④ In parallel, blood absorption and physical sealing by the gelatin layer support stable, sustained hemostasis. The schematic is illustrative and is not drawn to scale. Abbreviations: CHI-C, catechol-functionalized chitosan.

The present findings are plausible considering the bilayer architecture of InnoSEAL Plus DL (Figure 2). Unlike fibrin sealant patches, which rely on exogenous thrombin and fibrinogen to drive the terminal steps of the coagulation cascade [1,8], InnoSEAL Plus DL is composed of a CHI-C adhesive layer and a gelatin sealing layer that act in a complementary manner (Figure 2A) [35,37]. Upon contact with the bleeding hepatic surface, the CHI-C layer dissolves in blood and rapidly binds blood proteins through noncovalent catechol-mediated interactions to form an initial blood-insoluble hemostatic barrier. This barrier is subsequently reinforced both by covalent bonding between catechol groups and tissue and blood proteins and by chitosan-mediated aggregation of platelets and blood cells (Figure 2B) [35,37]. In parallel, the gelatin layer absorbs blood, provides physical sealing, and supports mechanical reinforcement during the early phase of hemostasis [1,25,37]. This multimodal mechanism is largely independent of the intact host coagulation cascade, a feature that was previously demonstrated under heparinized conditions in a preclinical model [36] and mechanistically distinguishes catechol-based platforms from fibrinogen- and thrombin-dependent fibrin sealants. The comparable hemostatic outcomes observed in this trial therefore support the translational potential of this non-fibrin, bioinspired biomaterial strategy for surgical bleeding control. This bilayer architecture also offers practical handling advantages. The blue CHI-C side provides a visual orientation cue similar to the yellow active side of TachoSil, and the non-adhesive gelatin side permits safe manipulation with forceps or gloved fingers, although the active surface adheres to wet instruments similarly to TachoSil. The patch is also sufficiently flexible to be rolled through a standard laparoscopic trocar and unfolded at the bleeding site, supporting its use in both open and laparoscopic hepatectomy.

The results should also be interpreted in the context of the trial setting. Hemostatic success within 3 min was achieved in all patients in both groups, a rate that is higher than those reported in historical studies of TachoSil [39,45,47,48]. This uniformly high success rate suggests a potential ceiling effect on the primary endpoint and warrants careful interpretation. This favorable result likely reflects several features of the present study, including rigorous intraoperative eligibility criteria restricted to persistent but controllable oozing after primary hemostasis, a standardized assessment schedule, and procedures performed by experienced hepatobiliary surgeons at high-volume centers. The 3 min hemostatic success rate observed for TachoSil is also consistent with the high success rate previously reported for it (48/49, 97.96%) in our prior randomized noninferiority trial of InnoSEAL Plus vs. TachoSil in hepatectomy patients conducted at the same institutions and by the same investigator group [38]. Consistent with this prior evidence, the 3 min hemostatic success rate was conservatively assumed to be 89.31% in both groups in the prespecified sample size calculation. Importantly, the potential ceiling effect does not preclude the noninferiority conclusion. Noninferiority was statistically supported by the prespecified CI criterion: the one-sided lower bound of the 97.5% CI for the absolute risk difference (−8.2 pp) was well above the prespecified margin of −19.4 pp. Concordant findings on the secondary endpoints, including time to hemostasis, 10 min hemostatic success, intraoperative rebleeding, and adverse events, provide additional support for equivalent intraoperative performance. Notably, the small numerical difference in time to hemostasis should be interpreted as evidence of comparable rapid hemostasis rather than clinically meaningful superiority of either device. However, because no intraoperative rebleeding events occurred in either group, this endpoint should be interpreted descriptively and does not allow meaningful comparison of rebleeding risk between treatments. Accordingly, the present findings are best interpreted as evidence that, under standardized conditions in a well-defined hepatectomy population with persistent oozing after primary hemostasis, InnoSEAL Plus DL performed comparably to TachoSil. These results should not be interpreted as evidence of superiority or of comparable efficacy across all bleeding severities and surgical environments.

This study also has several methodological strengths. The randomized, active-controlled, parallel-group design provided an appropriate framework for evaluating a topical hemostatic device against an established comparator [38,48]. The noninferiority margin was prespecified and clinically justified based on historical data [38,45], and the consistency of the findings across the per-protocol (PP) and intention-to-treat (ITT) populations supports the robustness of the primary conclusion. The potential impact of major protocol deviations, including prohibited tranexamic acid use, was also considered limited because these patients were excluded from the PP analysis, while the supportive ITT analysis, including all randomized and treated patients, yielded the same 3 min hemostatic success rate of 100.0% in both treatment groups. Although concomitant non-study procedures were more frequent in the InnoSEAL Plus DL group, these procedures were performed after study device application and mainly reflected treatment or prevention of hepatectomy-related postoperative adverse events. Therefore, this imbalance was considered unlikely to have influenced the prespecified 3 min hemostatic success assessment, but it should be considered when interpreting the higher total number of postoperative AEs and SAEs in the InnoSEAL Plus DL group. In addition, the study population was clinically relevant, as it comprised patients undergoing hepatectomy who required adjunctive topical hemostasis after conventional primary measures. Standardization of intraoperative evaluation time points and predefined efficacy and safety analysis sets further strengthened the internal validity of the trial.

From a clinical perspective, the adverse events observed in this trial were primarily perioperative events expected after hepatectomy, including gastrointestinal symptoms, procedural pain, pleural effusion, and a single case of intra-abdominal hematoma. Although the total number of AEs and SAEs was numerically higher in the InnoSEAL Plus DL group, the proportion of patients experiencing at least one AE was identical between groups, severe AEs occurred at the same frequency, and the between-group difference in SAEs was not statistically significant. Importantly, all SAEs were judged unrelated to the study devices, all patients with SAEs recovered without sequelae, and no device-related AEs, device-related SAEs, or deaths occurred. Therefore, the overall safety findings do not suggest a device-specific safety signal for InnoSEAL Plus DL in the studied adjunctive-use setting.

Nevertheless, several limitations should be acknowledged. First, this study was conducted at two high-volume centers in the Republic of Korea with substantial expertise in hepatobiliary surgery and transplantation. Thus, the generalizability to lower-volume institutions, non-Asian populations, and different healthcare settings remains to be verified. Second, the 3 min hemostatic success rate of 100.0% in both treatment groups suggests a potential ceiling effect, which limits the ability of the primary endpoint to discriminate small differences in hemostatic efficacy between devices. This pattern likely reflects the indication-restricted study population: enrollment was limited by protocol to patients with persistent oozing bleeding after completion of primary hemostasis, while patients with major bleeding were excluded because such bleeding requires definitive surgical control and falls outside the adjunctive-use setting evaluated in this trial (Section 2.1). Consequently, the present findings should not be extrapolated to more severe bleeding scenarios, patients with severe coagulopathy, or surgical fields beyond hepatectomy without dedicated evaluation in those settings. Future studies in more severe bleeding scenarios would require a different design, including standardized bleeding severity grading, stratification by bleeding intensity and source, predefined rescue hemostasis protocols, and clinically relevant endpoints such as time to hemostasis, need for additional hemostatic procedures, intraoperative blood loss, transfusion requirement, postoperative rebleeding, and safety outcomes. Although the hemostatic mechanism of InnoSEAL Plus DL may have potential relevance to other surgical settings involving persistent oozing after primary hemostasis, translation beyond hepatectomy will require dedicated studies that account for organ-specific tissue characteristics, bleeding patterns, local fluid environments, device handling, and postoperative safety outcomes. In addition, although the sample size was adequate for the prespecified noninferiority objective, the trial was not designed to assess superiority or to detect small between-group differences in uncommon adverse events. Patient-reported outcomes were not prespecified or collected. Therefore, postoperative pain scores, quality of life, recovery experience, and treatment satisfaction could not be compared between groups and should be incorporated in future studies. The trial included both open and laparoscopic hepatectomy and allowed primary hemostasis to be achieved using standard methods at each center, including suturing, ligation, vascular clips, argon beam coagulation, and electrocauterization. The distributions of key baseline and procedural factors relevant to potential subgroup effects were generally similar between groups, including cirrhosis (13.3% vs. 22.2%), open hepatectomy (28.9% vs. 26.7%), laparoscopic hepatectomy (71.1% vs. 73.3%), cautery (100.0% vs. 100.0%), sutures/ligation (95.6% vs. 100.0%), and vascular clips (71.1% vs. 73.3%). Although this reflects real-world hepatectomy practice, prespecified subgroup analyses were not performed, and the study was not designed or powered to evaluate treatment-by-subgroup interactions according to factors such as cirrhosis status, surgical approach, or specific primary hemostasis methods. Given the uniformly high 3 min hemostatic success rate in both treatment groups, post hoc subgroup analyses of the primary endpoint would have been statistically uninformative and potentially misleading. Future larger studies with prespecified subgroup analyses are warranted to evaluate the consistency of device performance across clinically relevant patient and procedural subgroups. Third, blinding of the operating surgeons was not feasible because the two hemostatic patches were visually distinguishable [38,48]. Moreover, the primary endpoint was visually assessed by the operating surgeon. To mitigate this risk, intraoperative eligibility was confirmed before randomization, device application and endpoint assessment were performed according to a prespecified protocol with predefined time points, and hemostatic success was judged using objective visual criteria, including no bleeding from the edges of the applied device and no bleeding through the device. Despite these efforts, because independent blinded adjudication or video-based central review was not performed, residual observer bias cannot be completely excluded and should be considered when interpreting the primary endpoint. Sponsor involvement should also be considered when interpreting the findings, because the study was funded by SCL Science Inc., the developer of InnoSEAL Plus DL, and two co-authors were employees of the sponsor. This potential source of bias was mitigated by MFDS and institutional review board approval, a prespecified protocol and statistical analysis plan, allocation concealment, predefined analysis populations, and reporting of both PP and ITT analyses. Finally, the follow-up was limited to 30 days and therefore did not permit assessment of long-term safety outcomes, including postoperative adhesion-related sequelae, delayed local tissue responses, late inflammatory reactions, or the full in vivo degradation profile of the material. Because InnoSEAL Plus DL is an absorbable bilayer biomaterial composed of gelatin and CHI-C, prior literature suggests that gelatin-based materials are generally absorbed over approximately 4–6 weeks, whereas chitosan-based materials undergo enzymatic degradation, mainly involving lysozyme and potentially chitinase or chitosanase, over several weeks to approximately 12 weeks [34,46,49]. Therefore, longer-term follow-up is needed to directly assess intra-abdominal device absorption, local tissue response, and potential adhesion-related sequelae.

From a translational perspective, these results suggest that a CHI-C/gelatin hemostatic platform may represent a clinically viable alternative to fibrin sealants in situations requiring rapid control of diffuse parenchymal oozing [25,32,35,37]. The combination of a CHI-C-based adhesive layer and a gelatin-based absorptive layer is consistent with current biomaterial strategies that seek to integrate tissue adhesion, fluid absorption, mechanical sealing, and blood-interactive hemostatic mechanisms within a single absorbable platform [49,50]. Because the material does not contain exogenous human coagulation factors, it may be of interest in clinical settings where synthetic or non-biological materials are preferred. In addition, this feature is particularly advantageous for patients with specific religious or ethical objections to human-derived products or in healthcare systems where cost-effectiveness favors synthetic alternatives. However, this trial was limited to oozing from the hepatic transection surface during hepatectomy. Further studies are warranted to evaluate the performance of InnoSEAL Plus DL in other surgical indications, in more challenging bleeding scenarios, and in patients with altered coagulation status. Larger studies with extended follow-up will also be important to further characterize long-term safety and postoperative outcomes. In summary, this trial provides evidence that InnoSEAL Plus DL is a safe and effective intra-abdominal hemostatic adjunct and supports its integration into contemporary hepatectomy practice as an alternative to established fibrin-based patches.

## 5. Conclusions

In this multicenter, randomized, single-blind, active-controlled, parallel-group noninferiority trial, InnoSEAL Plus DL, a bioinspired absorbable bilayer hemostatic patch composed of catechol-functionalized chitosan (CHI-C) and gelatin, was compared with TachoSil for the management of persistent oozing from the hepatic transection surface during hepatectomy. The 3 min hemostatic success rate was 100.0% in both groups, with the lower bound of the 95% CI for the absolute risk difference (−8.2 pp) well above the prespecified noninferiority margin of −19.4 pp, confirming the noninferiority of InnoSEAL Plus DL relative to TachoSil. The time to hemostasis was rapid and comparable between groups, no intraoperative rebleeding occurred, and the safety profile was consistent with events typically observed after hepatectomy, with no device-related serious adverse events or deaths.

These findings provide clinical evidence supporting the translational viability of catechol-based bioinspired biomaterials as a non-fibrin alternative for surgical hemostasis, with potential advantages in patients or settings in which human-derived coagulation factors are not preferred. Although the 30-day follow-up was sufficient to capture perioperative outcomes, longer-term studies and broader clinical indications, including more challenging bleeding scenarios, patients with impaired coagulation, and other surgical fields, are warranted to fully characterize the role of InnoSEAL Plus DL in contemporary surgical practice.

Overall, this trial supports the integration of InnoSEAL Plus DL into the available hemostatic options for hepatic surgery and contributes translational clinical evidence for the broader field of bioinspired adhesive biomaterials.

## Figures and Tables

**Table 1:** Baseline demographic and clinical characteristics of the ITT population.

Characteristic	InnoSEAL Plus DL (*N* = 45)	TachoSil (*N* = 45)
Age, years	46.5 ± 15.3	48.2 ± 13.0
Sex, n (%)		
Male	26 (57.8)	21 (46.7)
Female	19 (42.2)	24 (53.3)
Height, cm	166.7 ± 8.9	164.7 ± 8.4
Weight, kg	69.1 ± 9.8	65.4 ± 11.7
Indication for hepatectomy, n (%)		
Hepatocellular carcinoma	19 (42.2)	15 (33.3)
Intrahepatic cholangiocarcinoma	2 (4.4)	0 (0.0)
Metastatic liver disease	1 (2.2)	2 (4.4)
Donor hepatectomy	19 (42.2)	22 (48.9)
Other	4 (8.9)	6 (13.3)
Underlying liver disease, n (%)		
Hepatitis B	14 (31.1)	12 (26.7)
Hepatitis C	2 (4.4)	0 (0.0)
Cirrhosis	6 (13.3)	10 (22.2)
Alcohol-related liver disease	1 (2.2)	1 (2.2)
Nonalcoholic steatohepatitis	1 (2.2)	0 (0.0)
Comorbidities, n (%)		
Hypertension	11 (24.4)	13 (28.9)
Diabetes mellitus	8 (17.8)	5 (11.1)

Values are mean ± SD or n (%). Percentages were calculated within each treatment group. For underlying liver disease and comorbidities, multiple responses were permitted; therefore, percentages may not sum to 100%. No formal hypothesis testing of between-group differences was performed, in accordance with CONSORT guidelines. Abbreviations: ITT, intention-to-treat; SD, standard deviation.

**Table 2:** Procedural characteristics of the ITT population.

Variable	InnoSEAL Plus DL (*N* = 45)	TachoSil (*N* = 45)
Surgical approach, n (%)		
Open hepatectomy	13 (28.9)	12 (26.7)
Laparoscopic hepatectomy	32 (71.1)	33 (73.3)
Hepatic parenchymal morphology, n (%)		
Normal	37 (82.2)	38 (84.4)
Abnormal—cirrhosis	8 (17.8)	7 (15.6)
Resection type, n (%)		
Hemihepatectomy	27 (60.0)	31 (68.9)
Trisectionectomy	1 (2.2)	0 (0.0)
Extended hemihepatectomy	5 (11.1)	4 (8.9)
Sectionectomy	5 (11.1)	3 (6.7)
Segmentectomy	5 (11.1)	2 (4.4)
Wedge resection	2 (4.4)	4 (8.9)
Cyst excision	0 (0.0)	1 (2.2)
Primary hemostasis, n (%)		
Cautery	45 (100.0)	45 (100.0)
Sutures/ligation	43 (95.6)	45 (100.0)
Vascular clips	32 (71.1)	33 (73.3)

Values are n (%). Percentages were calculated within each treatment group. Primary hemostasis techniques were not mutually exclusive; therefore, percentages may not sum to 100%. No formal hypothesis testing of between-group differences was performed, in accordance with CONSORT guidelines. Abbreviations: ITT, intention-to-treat.

**Table 3:** Primary and secondary endpoints in the PP and ITT populations.

Endpoint	PP Population		ITT Population	
	InnoSEAL Plus DL (*N* = 43)	TachoSil (*N* = 41)	InnoSEAL Plus DL (*N* = 45)	TachoSil (*N* = 45)
**Primary endpoint**				
Hemostatic success within 3 min, n (%)	43 (100.0)	41 (100.0)	45 (100.0)	45 (100.0)
Risk difference (95% CI), pp	0.0 (−8.2–8.6) *		0.0 (−7.9–7.9) *	
Risk ratio (95% CI)	1.0 (0.9–1.1) ^§^		1.0 (0.9–1.1) ^§^	
**Secondary endpoints**				
Hemostatic success within 10 min, n (%)	43 (100.0)	41 (100.0)	45 (100.0)	45 (100.0)
Risk difference (95% CI), pp	0.0 (−8.2–8.6) *		0.0 (−7.9–7.9) *	
Risk ratio (95% CI)	1.0 (0.9–1.1) ^§^		1.0 (0.9–1.1) ^§^	
Time to hemostasis, min				
Mean ± SD	1.2 ± 0.4	1.3 ± 0.4	1.2 ± 0.4	1.3 ± 0.4
Median (range)	1.1 (0.8–3.0)	1.1 (1.0–3.0)	1.1 (0.8–3.0)	1.1 (1.0–3.0)
*p*-value	0.979 ^†^		0.942 ^†^	
Rebleeding rate, n (%)				
Yes	0 (0.0)	0 (0.0)	0 (0.0)	0 (0.0)
No	43 (100.0)	41 (100.0)	45 (100.0)	45 (100.0)
*p*-value	Not estimated ^‡^		Not estimated ^‡^	

Values are presented as n (%), mean ± SD, or median (range). * The 95% CIs for risk differences were calculated using the Newcombe–Wilson score method. ^§^ The 95% CIs for risk ratios were calculated using the exact binomial (Clopper–Pearson) method. ^†^ *p*-values were calculated using the Wilcoxon rank-sum test. ^‡^ *p*-values for rebleeding were not estimated because no events occurred in either group. Abbreviations: PP, per-protocol; ITT, intention-to-treat; N, total number of patients; min, minutes; n, number of patients with event; CI, confidence interval; pp, percentage points; SD, standard deviation.

**Table 4:** Summary of safety outcomes in the safety population.

Event	InnoSEAL Plus DL (*N* = 45)	TachoSil (*N* = 45)
**Adverse events (AEs)**		
Patients with ≥1 AE, n (%)	39 (86.7)	39 (86.7)
Total number of AEs, n	110	83
*p*-value	1.000 ^†^	
Severity, n (%)		
Mild	33 (73.3)	28 (62.2)
Moderate	16 (35.6)	14 (31.1)
Severe	2 (4.4)	2 (4.4)
Device-related AEs, n (%)	0 (0.0)	0 (0.0)
Deaths, n (%)	0 (0.0)	0 (0.0)
**Serious adverse events (SAEs)**		
Patients with ≥1 SAE, n (%)	4 (8.9)	2 (4.4)
Total number of SAEs, n	4	2
*p*-value	0.677 ^‡^	
SAEs by PT *, n (%)		
Pleural effusion	4 (8.9)	1 (2.2)
Intra-abdominal hematoma	0 (0.0)	1 (2.2)
Severity, n (%)		
Mild	0 (0.0)	0 (0.0)
Moderate	4 (8.9)	1 (2.2)
Severe	0 (0.0)	1 (2.2)
Outcome, n (%)		
Recovered without sequelae	4 (8.9)	2 (4.4)
Device-related SAEs, n (%)	0 (0.0)	0 (0.0)
Deaths, n (%)	0 (0.0)	0 (0.0)

Values are presented as n (%) or n. Percentages were calculated using the number of patients in each treatment group as the denominator. A single patient could appear in more than one row if multiple events of different types or severities were reported, but within each row, each patient was counted only once. ^†^ *p*-values for the comparison of the proportion of patients with ≥1 AE between groups, calculated using the chi-square test. ^‡^ *p*-values for the comparison of the proportion of patients with ≥1 SAE between groups, calculated using Fisher’s exact test. * SAEs were coded by Preferred Term (PT) using the Medical Dictionary for Regulatory Activities (MedDRA), version 26.1. Abbreviations: AE(s), adverse event(s); SAE(s), serious adverse event(s); PT, preferred term.

## Data Availability

The data presented in this study are available on reasonable request from the corresponding author. The data are not publicly available due to privacy and ethical restrictions related to the clinical trial participants.

## Supplementary Materials

The following supporting information can be downloaded at: https://www.mdpi.com/article/10.3390/biomedicines14051087/s1. Table S1: Eligibility criteria of participants.

## Abbreviations

The following abbreviations are used in this manuscript:

AE(s)	Adverse event(s)
CHI-C	Chitosan–catechol, catechol-functionalized chitosan
CI	Confidence interval
CONSORT	Consolidated Standards of Reporting Trials
FAS	Full analysis set
ITT	Intention-to-treat
MedDRA	Medical Dictionary for Regulatory Activities
MFDS	Ministry of Food and Drug Safety
POD	Postoperative day
PP	Per-protocol
pp	Percentage point
PT	Preferred term
SAE(s)	Serious adverse event(s)
SD	Standard deviation
TEAE(s)	Treatment-emergent adverse event(s)
