# Supplementary material for: Efficacy and Safety of a Bioinspired Chitosan–Catechol/Gelatin Hemostatic Patch vs. TachoSil in Hepatectomy: A Randomized Noninferiority Trial
Source: Biomedicines. 2026 May 12;14(5):1087. doi: 10.3390/biomedicines14051087 (PMC13204208; doi:10.3390/biomedicines14051087)
Supplement: Supplementary file 1 [file biomedicines-14-01087-s001.zip › biomedicines-4258335-supplementary.pdf]

Supplementary Table S1. Eligibility criteria of participants.

	**Inclusion Criteria**
1.	Age ≥ 19 years
2.	Planned hepatectomy for hepatocellular carcinoma, intrahepatic cholangiocarcinoma, intrahepatic cholelithiasis, metastatic liver cancer, or living-donor hepatic resection
3.	After completion of hepatectomy and standard primary hemostatic measures (e.g., suturing, ligation, vascular clips, argon beam coagulation, or electrocautery), the hepatic transection surface showed persistent oozing-type bleeding, defined as minor capillary bleeding from small vessels that did not require additional invasive hemostatic procedures during the operation
4.	Voluntary decision to participate in the trial with signed informed consent and willingness to comply with the study protocol
	**Exclusion Criteria**
1.	Presence of major bleeding after primary hemostasis, defined as spurting hemorrhage (typically arterial) or bleeding from major hepatic veins draining into the inferior vena cava
2.	History of cirrhosis and current portal hypertension
3.	History of liver transplantation or portal vein embolization
4.	Total bilirubin ≥ 2.5 mg/dL
5.	Aspartate aminotransferase (AST) or alanine aminotransferase (ALT) > 120 IU/L
6.	Severe coagulopathy, defined as international normalized ratio (INR) > 2.0 or platelet count $< 50 \times 10^9$/L
7.	Ongoing treatment with procoagulant agents or antifibrinolytic drugs
8.	Known immune-mediated disease
9.	Other conditions such as severe hepatic dysfunction, disseminated intravascular coagulation (DIC), hemolytic and/or hemorrhagic anemia, or autoimmune disease that, in the investigator’s judgment, precluded safe participation
10.	History of severe surgical complications
11.	Known hypersensitivity to any component of the test device or the comparator device
12.	Current drug abuse or alcohol dependence
13.	Requirement for emergency hepatectomy
14.	Pregnancy or breastfeeding
15.	Participants in another clinical trial within 1 month before screening that could interfere with the outcomes
16.	Any other reason for which the investigator considered participation inappropriate
